# Rice *Chalky Grain 5* regulates natural variation for grain quality under heat stress

**DOI:** 10.3389/fpls.2022.1026472

**Published:** 2022-10-11

**Authors:** Anil Kumar Nalini Chandran, Jaspreet Sandhu, Larissa Irvin, Puneet Paul, Balpreet K. Dhatt, Waseem Hussain, Tian Gao, Paul Staswick, Hongfeng Yu, Gota Morota, Harkamal Walia

**Affiliations:** ^1^ Department of Agronomy and Horticulture, University of Nebraska-Lincoln, Lincoln, NE, United States; ^2^ Rice Breeding Innovation Platform, International Rice Research Institute (IRRI), Los Banos, Philippines; ^3^ Department of Computer Science and Engineering, University of Nebraska-Lincoln, Lincoln, NE, United States; ^4^ Department of Animal and Poultry Sciences, Virginia Polytechnic Institute and State University, Blacksburg, VA, United States

**Keywords:** rice, grain, chalkiness, heat stress, GWAS - genome-wide association study, imaging

## Abstract

Heat stress occurring during rice (*Oryza sativa*) grain development reduces grain quality, which often manifests as increased grain chalkiness. Although the impact of heat stress on grain yield is well-studied, the genetic basis of rice grain quality under heat stress is less explored as quantifying grain quality is less tractable than grain yield. To address this, we used an image-based colorimetric assay (Red, R; and Green, G) for genome-wide association analysis to identify genetic loci underlying the phenotypic variation in rice grains exposed to heat stress. We found the R to G pixel ratio (RG) derived from mature grain images to be effective in distinguishing chalky grains from translucent grains derived from control (28/24°C) and heat stressed (36/32°C) plants. Our analysis yielded a novel gene, rice *Chalky Grain 5* (*OsCG5*) that regulates natural variation for grain chalkiness under heat stress. *OsCG5* encodes a grain-specific, expressed protein of unknown function. Accessions with lower transcript abundance of *OsCG5* exhibit higher chalkiness, which correlates with higher RG values under stress. These findings are supported by increased chalkiness of *OsCG5* knock-out (KO) mutants relative to wildtype (WT) under heat stress. Grains from plants overexpressing *OsCG5* are less chalky than KOs but comparable to WT under heat stress. Compared to WT and OE, KO mutants exhibit greater heat sensitivity for grain size and weight relative to controls. Collectively, these results show that the natural variation at *OsCG5* may contribute towards rice grain quality under heat stress.

## Introduction

Heat stress (HS) poses a serious threat to agriculture production and food security. Maximum daytime temperature that exceeds 33°C during reproductive development affects pollen viability and multiple yield parameters (Hatfield and Prueger, 2015). In the absence of genetic improvement of crops for enhanced heat resilience, every 1°C temperature increment is predicted to result in yield loss of 3.2% for rice, 6% for wheat, 7.4% for maize, and 3.1% for soybean (Zhao et al., 2017). Rice yield loss is particularly detrimental as it serves as a major dietary source for nearly 3.5 billion people (Wing et al., 2018). Besides yield, HS occurring during grain development also reduces rice grain quality (Ishimaru et al., 2009; Krishnan et al., 2011; Sreenivasulu et al., 2015; Nakata et al., 2017; Shi et al., 2017; Wada et al., 2019; Zhen et al., 2019; Paul et al., 2020). These yield and quality constraints highlight the need for developing heat resilient rice cultivars (Fragkostefanakis et al., 2015; Geange et al., 2021). Various crop improvement programs have used genome-wide association studies (GWAS), genetic mapping or reverse genetics approaches to characterize major QTLs for rice yield and grain size (Huang et al., 2013). However, our understanding of the genetic basis of grain yield and especially quality under HS is still limited due to challenges in imposing a targeted HS for large number of accessions (Xu et al., 2021). The common prioritization of grain yield over grain quality in breeding programs has also led to development of many varieties that are preferred by farmers for their yield, but not by consumers. Climate driven higher temperature during grain development is predicted to further exacerbate this problem (Morita et al., 2016).

Rice quality traits are highly correlated with the market price (Cuevas et al., 2016; Custodio et al., 2019; Yang et al., 2021). For instance, milled grains are graded on their percentage of chalkiness, chalky grains being more prone to breakage in the milling process due to the lower intrinsic grain strength caused by airspace among the abnormal starch granules (Misra et al., 2021). Grain chalkiness is a polygenic trait identified as opaque white discoloration of the translucent endosperm (Armstrong et al., 2019). HS occurring during grain development triggers an increase in grain chalkiness (Tashiro and Wardlaw, 1991; Yamakawa et al., 2007; Fitzgerald and Resurreccion, 2009; Masutomi et al., 2015; Morita et al., 2016; Paul et al., 2020). HS causes misregulation of genes that control starch and storage protein metabolic pathways (Liu et al., 2010; Yamakawa & Hakata, 2010; Hakata et al., 2012; Kaneko et al., 2016; Ishimaru et al., 2019; Gann et al., 2021; Wang et al., 2021). For instance, *Chalk5* and *UGPase1* are two of the genes known to contribute to variation in grain chalkiness (Li et al., 2014a; Woo et al., 2008). Other grain quality genes affected by HS include transcription factors and genes regulating phytohormone homeostasis (Zhu et al., 2011; Wang et al., 2013; Kaneko et al., 2016; Zhang et al., 2018; Wang et al., 2020; Xu et al., 2020; Baysal et al., 2020). However, the extent to which the determinants of natural variation for grain quality under normal temperatures will be involved in grain quality variation under HS is not known.

Grain properties such as chalkiness, color, and shape have been quantified using imaging systems. For instance, support vector machine (SVM) and digital image processing have been used to analyze grain chalkiness and detect structural abnormalities in rice (Yoshioka et al., 2007; Sun et al., 2014; Chen et al., 2019; Ma et al., 2020; Aznan et al., 2021). Significant improvement to these approaches, deep learning-based supervised segmentation methods can estimate HS-induced grain chalkiness (Wang et al., 2022). Apart from area-based, two-dimensional imaging systems, the three-dimensional high-resolution X-ray microcomputed tomography technique has also been utilized as a volume-based approach to accurately quantify grain chalkiness (Su and Xiao, 2020). Hyperspectral imaging system has also been recently used to analyze grain quality (Caporaso et al., 2018; Armstrong et al., 2019; Feng et al., 2019; Gao et al., 2021). For instance, recent studies have combined hyperspectral imaging and genetic association studies to identify several loci associated with grain chalkiness (Barnaby et al., 2020; Xiao et al., 2022). However, a similar approach combining imaging and genetic association analysis has not been explored for grain quality determination under HS.

Conventional phenotypic evaluation of grain chalkiness adopts commercial grain analytical scanners and imaging systems (Qiu et al., 2015; Marschalek et al., 2017; Misra et al., 2021). These typically require large grain quantities that are intended for field-scale experiments. However, conducting precisely timed HS experiments for a diverse set of accessions with varying flowering time is not practical in the field environment. Rather, controlled environment conditions combined with image-based software that can rapidly quantify the optical properties of small quantities of grains is preferred for mapping grain traits from a diverse set of accessions (Velesaca et al., 2021). *SeedExtractor*, an open source imaging software, can accurately measure grain colors in three broadband color intensities, Red (R), Green (G), and Blue (B) (Zhu et al., 2021a). Each pixel in digital images in RGB format ranges from 0-0-0 to 255-255-255 and produce a single-color value for that pixel in the image (Dell’Aquila, 2006; Elmasry et al., 2019). RGB intensities associated with grain pixel are then used to analyze changes in grain properties. The ratio of R to G spectral reflectance (*R*_RED_ *: R*_GREEN_) is a robust index to quantify leaf pigmentation patterns (Gamon and Surfus, 1999). However, the significance of RGB channel intensities and their ratios in the context of grain chalkiness is not reported. In this study, we examined the potential of using the R to G pixel ratio (RG) as an indicator of grain quality. To achieve this, we first imposed a HS treatment on a set of accessions from rice diversity panel 1 (RDP1) (Eizenga et al., 2014). Using grains derived from these treatments for imaging, we obtained RG values as a derived phenotypic trait under control and HS for genome-wide association (GWA) analysis. We have identified a candidate gene, *rice chalky grain 5* (*OsCG5*) associated with a significant locus on chromosome 5. Higher *OsCG5* transcript level negatively correlates with grain chalkiness under HS. Grains of mutant plants deficient in *OsCG5* have greater sensitivity to HS and those from overexpression plants are less sensitive to HS.

## Material and methods

### Plant material and growth conditions

We selected 229 accessions from RDP1 representing different sub-populations of rice germplasm for evaluating the phenotypic variation in grain quality in response to HS (Zhao et al., 2011; Eizenga et al., 2014; McCouch et al., 2016). Accessions selected from RDP1 panel represent five major sub-populations spanning diverse geographical origins, including 41 indica, 55 temperate japonica, 50 tropical japonica, 39 aus, 7 aromatic, 25 admixed indica or japonica and a set of 12 accessions lacking subspecies information (**Figure S1**; **Table S1**). Dehulled rice grains, sterilized with bleach (40% v/v) for 40 min, and soaked in sterile water overnight, were germinated on half-strength Murashige and Skoog (MS) media for 2d in the dark, followed by 1d growth in light. Seedlings transplanted in 10 cm square pots that contained natural soil mix were grown under a controlled greenhouse diurnal setting with temperature 28/24 ± 1°C, light/dark 16/8 h, and relative humidity of 55-60%. Spikelets were marked to record the flowering time, and half of the plants (2-8 replicates per accession for each treatment) were given HS treatment (36/32 ± 1°C) 1d after flowering of marked spikelets. HS condition was maintained for 5 d, and treated plants were moved back to the control (28/24°C) greenhouse until maturity. Marked mature dehulled grains harvested from both control and HS treated plants were used for grain image analysis.

### Mature seed morphometric and colorimetric analysis

Harvested panicles from control and HS treated plants were dried for two weeks (28°C), and dehulled marked grains were collected for imaging. Dehulled grains were scanned using Epson Expression 12000 XL scanner (Epson America Inc., Los Alamitos, CA, USA) at 600 dpi resolution. Scanned images were processed using a MATLAB application, *SeedExtractor* (Zhu et al., 2021a). After removing the grain shape outliers and filtering for normality, the adjusted mean for each accession across replicates were obtained with the statistical model as described previously (Zhu et al., 2021a).

### Genome-wide association study (GWAS)

A 700K high-density rice array marker dataset was used to run the GWAS (McCouch et al., 2016). In total, 411,066 SNPs were retained after filtering for missing data (< 20%) and minor allele frequency (< 5%). The population structure of the studied accessions was assessed using principal component analysis (PCA) on the constructed genomic relationship matrix (Zheng et al., 2012) (**Figure S1**). GWAS was conducted in rrBLUP R package (Endelman, 2011) using the linear mixed model described earlier (Dhatt et al., 2021). SNP markers were declared significant using the *P*-value threshold of –log10(*P*) > 6.5, based on method of Li and Ji (2005) using effective number of markers (Li and Ji, 2005; Hussain et al., 2020). Manhattan plot and Q-Q plot were created using R package qqman (Turner, 2018). Phenotypic variance (*R^2^*) explained by each SNP was estimated using the *mixed.solve ()* function from the rrBLUP R package (Endelman, 2011) with SNP having variance equal to *Kσ^2^u*, where ***K*** is the design matrix of SNP and *u* is the random effect of the SNP. Additionally, *R^2^* explained by the locus having all the significant SNPs was estimated using BGLR R package (Pérez and De Los Campos, 2014). For this, all the SNPs were fitted jointly accounting the LD between the markers *via* a genomic restricted maximum likelihood method (Dhatt et al., 2021).

### Vector construction and transgenics generation

We generated mutant and overexpression lines of *OsCG5* (*LOC_Os05g40850*) associated with SNP-5.23896968 at the position 23,959,548 bp (chr 5) to investigate the genetic basis of grain chalkiness. For *OsCG5* CRISPR-Cas9 mutants, the single-guide RNAs (sgRNAs) designed using CRISPR-P 2.0 (http://crispr.hzau.edu.cn/CRISPR/) was cloned as described by Lowder et al. (2015) (Lei et al., 2014; Lowder et al., 2015). The single-guide sequence cloned in pYPQ141C (using Esp3I/BsmBI site) was recombined with pANIC6B and pYPQ167 (Cas9) using LR-clonase. Overexpression construct for *OsCG5* was generated using Gateway cloning system. For this, the genic region and ~2kb upstream of *OsCG5* amplified from Kitaake DNA using Phusion High Fidility PCR master mix (ThermoScientific, USA) was cloned in pENTR-D-Topo vector (ThermoScientific, USA) to get an entry clone. The entry clone was recombined with modified pMDC99 that contained NOS terminator (Curtis and Grossniklaus, 2003; Campbell et al., 2017) to construct the destination overexpression. For generating stable GUS lines, *OsCG5* promoter (~2 kb kb upstream of the start codon) amplified from Kitaake DNA was cloned into pENTR-D-Topo vector and then recombined with pMDC163 to get destination clone with GUS reporter. These destination constructs were then transformed into Agrobacterium tumefaciens strain EHA105, which was used for rice callus transformation (Cheng et al., 1998; Chen et al., 2016). For CRISPR-Cas9 lines, T1 plants were screened for the presence of Cas9 construct using GUS-based screening assay. The DNA extracted from plants lacking the Cas9 construct was used to screen for the presence of a mutation using Sanger sequencing. Homozygous plants from T3 or later generations were used for phenotypic evaluation. For overexpression lines, homozygous plants were used to confirm the overexpression of *OsCG5* using qPCR assay. For GUS assay, different plant tissues were stained with GUS solution as described previously (Schmitz et al., 2015). Primers used in the study are listed in **Table S2**.

### Phenotypic evaluation of grains from CRISPR-Cas9 and Overexpression transgenic plants

For analyzing the HS response of grains from transgenic plants and Kitaake (WT), spikelets were marked at flowering, and plants were exposed to either HS 1d after flowering (5d HS, 36/32°C) and returned to control condition or grown throughout in control greenhouse. At maturity, marked, dehulled grains were used for grain size and colorimetric analysis. In total, 6-7 plants for each genotype per treatment were used for phenotypic analysis (**Table S3**). Cumulatively, we used 3,988 marked grains from different genotypes and treatments for this analysis.

### Hyperspectral imaging of grains from transgenic plants

We measured hyperspectral reflectance (600-1700 nm) of grains from transgenic lines using the *HyperSeed* imaging platform (Gao et al., 2021). Briefly, grains from control and HS groups were placed on a constantly moving platform and scanned by a hyperspectral camera (Micro-Hyperspec Imaging Sensors, Extended VNIR version, Headwall Photonics, Fitchburg, MA, USA) with Exposure Time and Frame Period set to 12 ms and 18 ms, respectively. The images were captured in the form of three-dimensional (x, y, λ) hypercubes where x, y represented the position of the pixel in spatial dimensions, and λ referred to the index of wavelength in spectral dimension. Then the images were preprocessed by removing 5% of bands at the beginning and end of the spectrum for better accuracy and calibrated using dark and white references. Afterward, these images were further processed using a two-step grain segmentation algorithm to extract the grain spectra and remove the background. Spectral reflectance of grains from the same plant was averaged along spatial dimensions for further analysis.

### Statistical analysis of grain RG, morphometrics and gene expression data

The significance level for grain RG and morphometrics data [grain length, grain width, grain area, and single grain weight (sgw)] was determined using two-way ANOVA. Students t-test was used to test for statistical significance for gene expression within and between allelic groups. PCA was used to inspect the RDP1 population structure using the R packages FactoMineR and factoextra (Lê et al., 2008). Pairwise Pearson correlation coefficient (PCC) of *OsCG5* with all other grain expressed genes were calculated using rcorr function with the Pearson option in Hmisc R package (Harrell, 2014). All statistical analyses in this study were performed in the R environment (R Core Team, 2019).

## Results

### Phenotypic variation in heat stress response of grain colorimetric parameters

**Figure 1:**
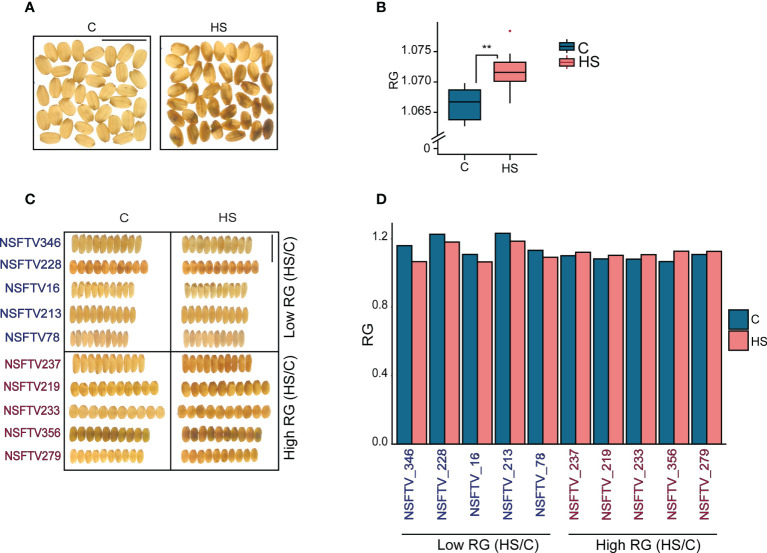
Relation between variation in the ratio of grain R and G pixel intensity (RG) values and grain chalkiness in response to heat stress (HS). **(A)** Light box images of control and HS-treated rice (cv. Kitaake) grains showing the difference in grain chalkiness. **(B)** RG values of control and HS-treated grains **(C)** Light-box images of 10 selected RDP1 accessions with a lower and higher percentage change for RG (HS/Control) **(D)** RG values of grains shown in **(C)** C and HS indicate control and heat stress, respectively. ** indicates the significance of a t-test with *P*<0.05. Scale bar=1cm.

To elucidate the phenotypic variation in grain quality in response to HS, we exposed 229 accessions from the RDP1 to 5 d of HS (36/32 ± 1°C) treatment beginning at 1d after flowering (DAF) and a corresponding set to control (28/24 ± 1°C) treatment. Flowering spikelets were individually marked on the day of fertilization and tracked during the course of the HS treatment. We collected the mature, marked grains and dehulled them before scanning for grain color (R, G and B) pixels using the *SeedExtractor* (Zhu et al., 2021a). We only used R and G channel colors for examining the impact of HS on the R and G pixel intensities from control and HS treatment grains. We sought to determine if the ratio of R to G pixel intensities (RG) for grains can be used as proxy for grain chalkiness caused by HS treatment. Visual examination of grains indicates that even a transient HS treatment increases grain chalkiness. To test this, we measured the RG values of translucent and chalky grains obtained from control and HS treated plants of Kitaake cv, respectively (**Figure 1A**). The RG value of HS-treated chalky grains were significantly higher than the translucent control grains (**Figure 1B**). A similar measurement for the RDP1 accessions had a range of grain RG (1.03 to 1.57), with mean RG values under control and HS to be 1.13 and 1.12, respectively (**Table S4**). The RDP1 mean values for RG for control and HS were similar due to the fact that grains from many accessions vary in opposite directions in their response to HS.

We next examined whether the HS sensitivity for the RG trait among RDP1 accessions correlated with chalkiness for several diverse accessions and obtained the percentage change of their RG values under HS (HS/Control) (**Figure 1**; **Table S4**). We visually confirmed that the accessions with higher HS/Control for RG values generally had higher chalkiness under HS compared to corresponding controls and, hence, were considered more heat-sensitive in grain quality context (**Figures 1C** **, D**). Conversely, accessions with lower HS/Control for RG exhibited relatively lower grain chalkiness in general. These results suggest that RG values are associated with grain chalkiness under HS among the diverse accessions, although there are some accessions where this relationship does not hold true (**Figures 1C** **, D**).

### Genome-wide association analysis for loci associated with RG

**Figure 2:**
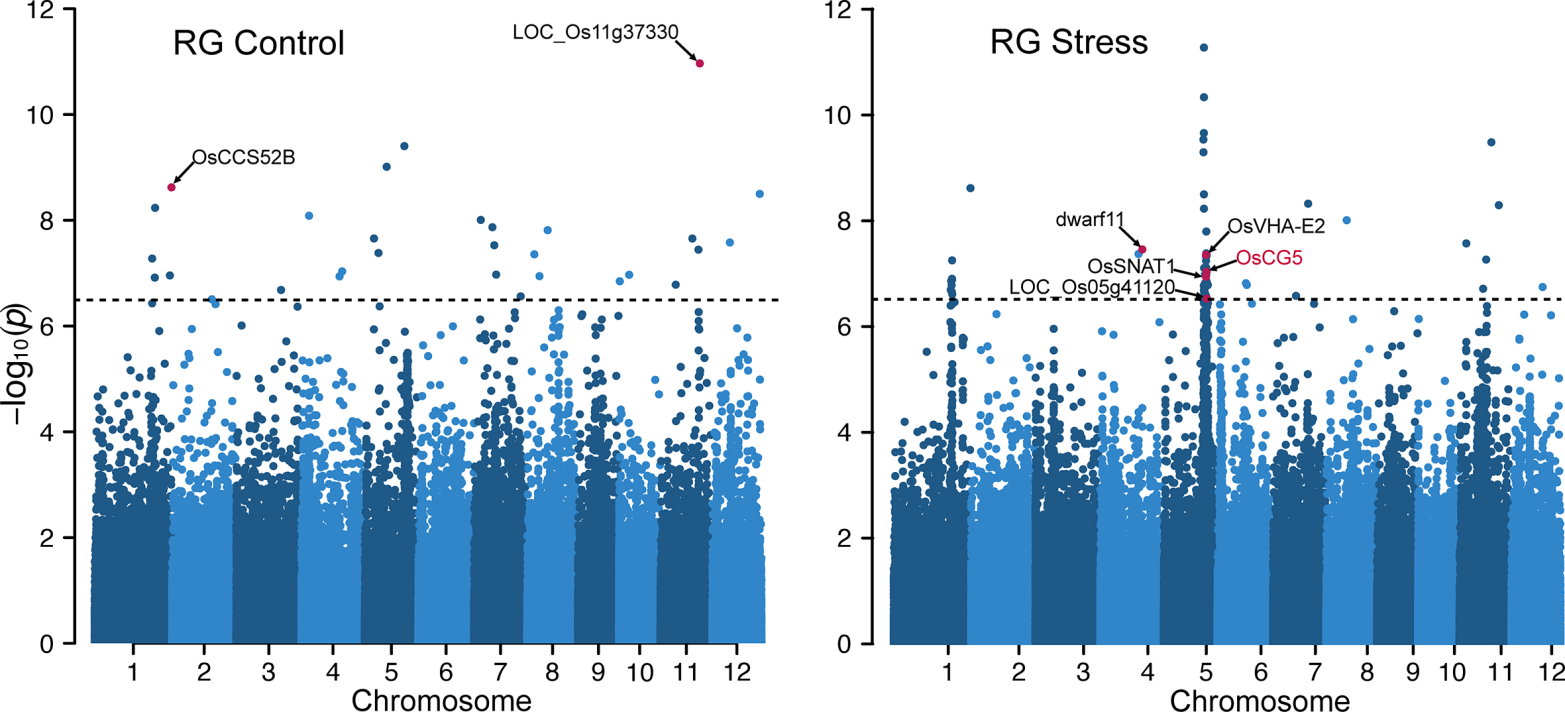
Manhattan plots of genome-wide association studies for the ratio of grain R and G pixel intensity values under control and heat stress. The black dotted horizontal line represents the genome- wide significance threshold (–log10(*P*) > 6.5). SNPs associated with significant genes are highlighted.

Given the association between RG values and chalkiness, we incorporated the RG values as a phenotypic trait to dissect the genetic basis of grain chalkiness. We conducted independent GWA analysis for control and HS treatments. The GWA analysis identified 106 significant SNPs that are strongly associated (–log_10_(*P*) > 6.5) with RG values (**Figure 2**). Of these 30 SNPs were detected from analysis of control condition grains and 76 SNPs from the HS treatment. Only seven SNPs, underlying seven peaks were detected in both conditions (**Table S5**). Under control conditions SNP-11.21577974 on Chr 11 was the most significant (*P*=10.9) and was also detected under HS (*P*=8.28). This SNP localizes to the second intronic region of a pentatricopeptide repeat domain (PPR) protein-coding gene (*LOC_Os11g37330*). A mutant of another gene (non-homolog) from this domain family in Chr 11, *small kernel 1* (*LOC_Os11g10740*), is involved in grain development in rice and maize, and has a chalky and shrunken phenotype (Li et al., 2014b). We also identified SNPs that co-localize to genes functioning in grain development with a potential auxiliary role in chalkiness. For instance, SNP-1.42949271 (Control *P*=8.62, HS *P*=8.60) is located within a cell cycle switch 52B gene *OsCCS52B (LOC_Os01g74146)* that controls cell size and regulates endoreduplication to determine the grain size (Su’udi et al., 2012). Although it did not meet the stringent *P*-value cutoff, SNP-5.5143433 on Chr 5 appeared in both conditions (Control *P*=5.94, HS *P*=5.83) and is located 171bp upstream of an expressed protein, *LOC_Os05g09200*. Notably, *LOC_Os05g09200* has been proposed to be a regulator of grain chalkiness based on a targeted-gene association study (Misra et al., 2019).

Our analysis also identified SNPs that were either specific to HS treatment or were more significant under HS. For instance, a HS-specific SNP-4.23303276 (HS *P*=7.74) was found to be associated with a brassinosteroid biosynthesis gene *dwarf11* (*LOC_Os04g39430*) that regulates grain length (Tanabe et al., 2005). A frame shift mutation of *dwarf11* results in a notched belly phenotype with higher grain chalkiness (Tong et al., 2018). Given the detection of several genes with grains related functions, we mined all genes associated with significant SNPs under control and HS. Our selection criterion involved genes within 20 kb (10 kb upstream and downstream) of the most significant SNP for each peak, resulting in a list of 506 non-redundant genes (**Table S5**). We further filtered these genes based on their expression in developing grain using public dataset (GSE6893) and identified 10 genes that are preferentially expressed in grains. For the genes associated with SNP detected under HS treatment, we examined their expression in a comparable HS treatment in a public dataset (Sandhu et al., 2021). We identified 18 genes to be differentially expressed in response to HS (**Table S5**). As these genes are expressed in developing grains, they have a higher likelihood of impacting grain quality.

We detected a significant HS-specific peak on Chr 5, which spanned coordinates 22.2 to 24.86 Mb (RGAP V7). Cumulative phenotypic variance explained by the SNPs populating this region under HS and control was 0.46 and 0.22, respectively. Among these, SNP-5.23564097 is located upstream of a vacuolar H+ ATPase (*OsVHA-E2*; *LOC_Os05g40230*) whose isoform subunit *OsVHA-E1* traffics grain storage proteins and grains from the mutant plants have a floury appearance (Zhu et al., 2021b). SNP-5.23599535 is located downstream of serotonin N-acetyltransferase coding gene, *OsSNAT1*. Transgenic plants overexpressing *OsSNAT1* were shown to enhance grain yield due to increased panicle number per plant (Lee and Back, 2017). Endoplasmic reticulum (ER) stress induced by HS lead to floury or shrunken grain phenotype (Qian et al., 2015). Notably, ER compartment protein-coding gene *LOC_Os05g41120* associated with SNP-5.24040516 has been shown to have higher transcript abundance in developing grain (7 DAF) under ER stress (Oono et al., 2010). Our analysis of the expression profiles from developing grains showed that, out of the 216 genes associated with this prominent peak on Chr 5, only 13 (13/216) are HS responsive (**Table S5**). From these 13 genes, three genes are preferentially expressed in developing grains. Of them, *LOC_Os05g40790* is one of the five CCR4-NOT transcription factors in rice and *LOC_Os05g38530* is a member of the DnaK gene family (*LOC_Os05g38530*). The third gene encodes for an expressed protein (*LOC_Os05g40850*) with highest expression in developing grains (**Figure S2A**). *LOC_Os05g38530* is annotated as a member of the heat shock protein (HSP) 70 family (Jung et al., 2013). In contrast, *LOC_Os05g40850* is a single copy rice gene. Based on this cumulative analysis, we considered these three genes to be high priority candidates for regulating variation in grain chalkiness under HS at this locus.

**Figure 3:**
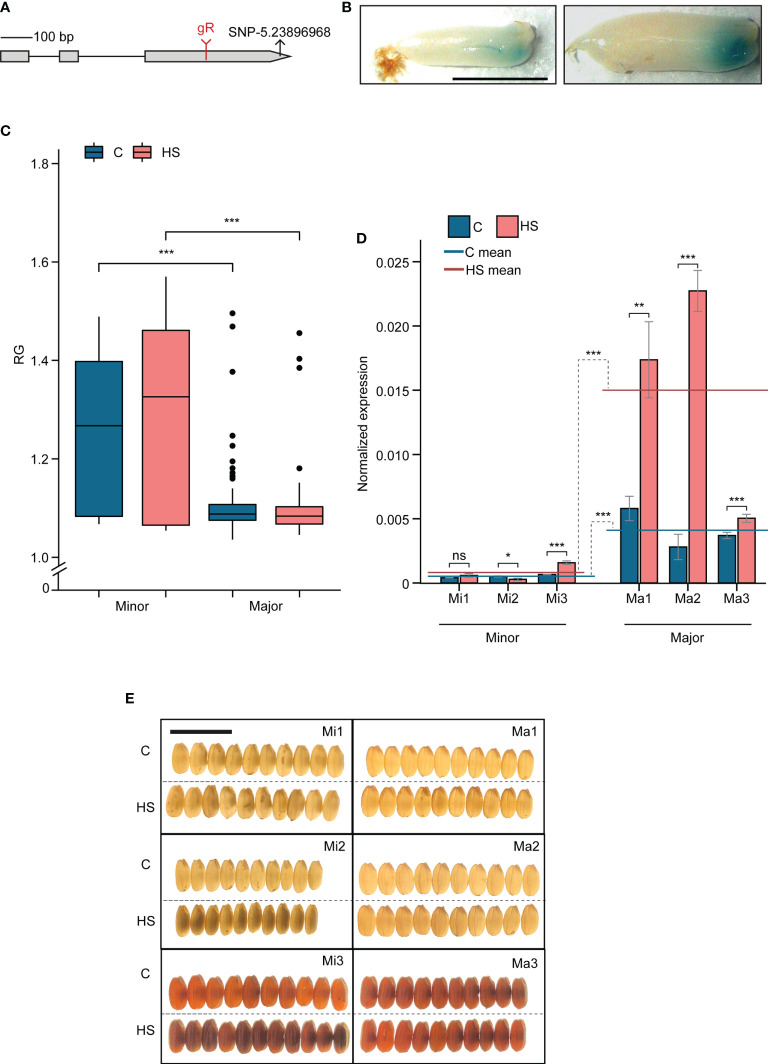
Grain chalkiness in *chalky grain 5* (*OsCG5*) allelic variants under heat stress (HS) is regulated by its transcript abundance **(A)** The structure of the *OsCG5* gene. The position of guide RNA (gR) and SNP-5.23896968 are labeled in red and black symbols, respectively. **(B)** Promoter-GUS expression of *OsCG5* in developing endosperm at 3 and 4 days after fertilization (DAF). Scale bar = 0.25cm **(C)** Distribution of the ratio of grain R and G pixel intensity (RG) of major and minor allelic accessions (Ma and Mi, respectively) under control and HS **(D)** RT-PCR based transcript estimation of *OsCG5* in 2 DAF old grains of Ma and Mi under control and HS. Ma1-NSFTV 113; Ma2- NSFTV 333; Ma3- NSFTV 255; Mi1-NSFTV 19; Mi2- NSFTV 33; Mi3- NSFTV 345. Scale bar=1 cm. **(E)** Lightbox images of grains from Ma and Mi whose expression of *OsCG5* transcript was estimated in **(D)** C and HS indicate control and heat stress, respectively. Significance level for t-test, *P<0.05; **P<0.01; ***P<0.001; ns, non-significant.

Given the early grain-specific expression that coincides with the HS treatment window and its HS response, we decided to determine if *LOC_Os05g40850* (named, *OsCG5*) regulates variation in grain chalkiness under HS (**Figure S2**). *OsCG5* carries a significant SNP-5.23896968 (Control *P*=5.25, HS *P*=7.02) within its exonic region (**Figure 3A**) and is located 1.4 Mb downstream of lead SNP (SNP-5.22423360) on Chr 5 under HS. Sequence homology search revealed no significant orthologues for this gene of unknown function. To confirm developing grain-specific expression of this gene, we analyzed *OsCG5* promoter-beta-glucuronidase (GUS) lines (*pOSCG5*::GUS) and found that GUS signal was restricted to the lower part of grains at 3 and 4 DAF (**Figure 3B**). Consistent with the expression patterns observed from public datasets, GUS activity was not detected in other developmental stages or tissues (**Figure S3**). HS increases transcript abundance of *OsCG5* in developing grains at 2 DAF (**Figure S2B**).

### Allelic variation in *OsCG5* expression correlates with grain chalkiness under heat stress

We analyzed the distribution of SNP-5.23896968 among RDP1 accessions and found that 90% of accessions contain the “G” allele (referred to as the major allele) and 10% of accessions have the “T” allele (referred to as the minor allele). Overall, the major allelic group shows lower RG values than minor accessions (**Figure 3C**). However, it should be noted that there is variability in RG values for accessions of the minor allele and some accessions for each allelic group have values that are equivalent to the highest and lowest values in the contrasting allele group (**Figure 3C**). This is expected given that grain chalkiness is a multigenic trait and also exhibits variation within the same panicle. We investigated if differential transcript abundance of *OsCG5* could be the basis of the phenotypic difference between the allelic groups. For this, we randomly selected three accessions from each allelic group and measured the expression of *OsCG5* in developing control and HS-treated grains at 2 DAF (1 day after stress), with HS initiated at 1 DAF (**Figure 3D**). We also evaluated grain chalkiness for these accessions by placing the grains on a light box (**Figure 3E**). We found that major allelic accessions (Ma) had relatively higher expression of *OsCG5* under both control and HS treatment. Further, Ma1 and Ma2 showed higher accumulation of *OsCG5* transcript in response to HS when compared to corresponding controls. Induction level of *OsCG5* transcript in Ma3 was lower compared to Ma1 and Ma2. Among minor allelic accessions (Mi), transcript abundance of *OsCG5* under HS was significantly reduced for Mi2 and increased for Mi3. For Mi1, transcript abundance of *OsCG5* did not change under HS compared to control. The average transcript abundance of *OsCG5* in Mi under HS was not significantly different than the corresponding average under control. In contrast, average transcript abundance of *OsCG5* in the three Ma under HS were significantly higher than control (3.7-fold; *P*<0.001). The Mi also exhibited higher levels of chalkiness than the Ma under HS (**Figure 3E**). These data suggest that transcript abundance of *OsCG5* could be positively associated with grain quality under HS.

### *OsCG5* knockouts are more sensitive to heat stress

**Figure 4:**
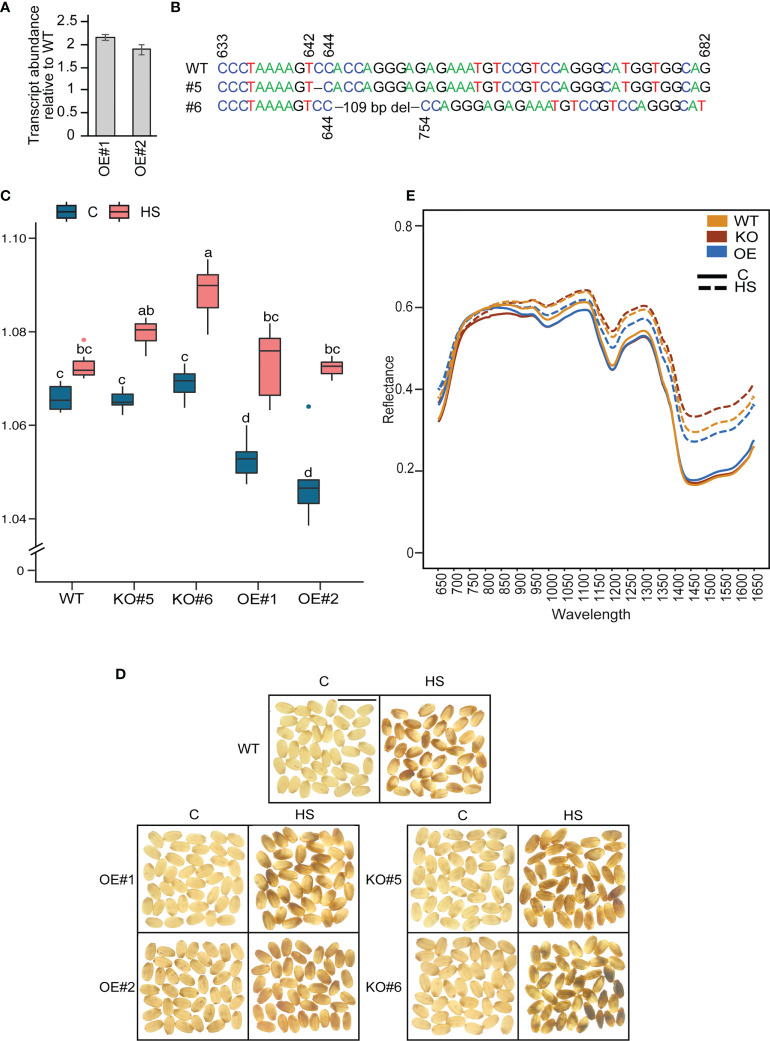
Characterization of the function of *chalky grain 5* (*OsCG5*) in grain chalkiness using CRISPR-Cas9 knockout (KO) and Overexpression (OE) lines. **(A)** RT-PCR assay showing higher transcript abundance of *OsCG5* in OE lines relative to WT rice (cv. Kitaake). **(B)** Positions of Cas9 deletions in KO#5 and KO#6 lines (1 bp and 109 bp, respectively). **(C)** Distribution of the ratio of grain R and G pixel intensity values in WT, KO and OE genotypes under control and heat stress (HS). The significance was estimated using two-way ANOVA. *N* = 7-8 plants. Scale bar=1 cm. **(D)** Phenotypic difference in grain chalkiness for WT, KO and OE under control and HS. Scale bar=1 cm. **(E)** Hyperspectral reflectance of grains from WT, KO and OE genotypes at wavelength range 650-1650 nm under control and HS. C and HS indicate control and heat stress, respectively.

To determine the role of *OsCG5* in regulating the grain quality under HS, we generated native (~ 2kb upstream) promoter-overexpression (OE) and CRISPR-Cas9 (CR)-based knockout (KO) mutants in cv Kitaake, which contains the “G” allele for SNP-5.23896968 and hence belongs to the major allelic group. Ma have higher transcript abundance of *OsCG5* and are less sensitive to HS than Mi. Therefore, we hypothesized that knocking-out *OsCG5* in a major allelic background will render it more sensitive to HS and cause higher RG values and chalkier grains than WT. We obtained two OE (OE1 and OE2) and two homozygous KO mutants (KO#5, KO#6) (**Figure 4**). Transcript abundance of *OsCG5* in the native promoter-driven OE lines is 2-fold higher relative to WT at 3 DAF grains (**Figure 4A**). The homozygous mutants KO#5 and KO#6, have 1 bp and 109 bp deletions in their target region, respectively (**Figure 4B**). KO mutants have reduced *OsCG5* transcript abundance relative to WT at 3 DAF grains (**Figure S4**). Under control conditions, OE grains showed a lower RG than KO and WT (**Figure 4C**). WT control grains had an RG similar to KO#5 and KO#6. However, light box imaging did not show a clear difference in appearance among the grains from three genetic backgrounds grown under control conditions. Under HS, grain RG values increased from their respective controls for all genotypes. KO#6 had higher RG values than OE and WT under HS. However, the RG value for KO#5 was not significantly different from WT under HS. This could be likely due to the 1 bp deletion in KO#5 compared to a large deletion in KO#6. Consistent with higher grain RG values observed under HS, KO#6 also showed higher chalkiness under HS than OE and WT (**Figure 4D**). However, overexpressing *OsCG5* did not result in decreased chalkiness under HS. These observations show that *OsCG5* positively contributes to grain quality under HS.

### Hyperspectral reflectance of grains from different genotypes corroborates the grain chalkiness quantified under HS

Grain chalkiness has recently been estimated using hyperspectral scanning in rice (Barnaby et al., 2020). Therefore, we *OsCG5* extended the mutant characterization by analyzing the hyperspectral reflectance of grains from WT, OE and KO lines to understand the variation in spectral reflectance (lines) of grains with different chalkiness levels (**Figure 4E**). Under control, spectral reflectance of OE, KO and WT grains was indistinguishable. Compared to control, HS-treated grains had higher spectral reflectance in 850-1650 nm range for all the genotypes. Genotypic difference under HS were primarily observed in the 1400-1650 nm range, where WT spectral reflectance was lower than KO and higher than OE. HS-treated KO grains showed highest spectral reflectance and showed maximum deviation from corresponding controls in this wavelength range, which may be indicative of higher grain chalkiness observed in KO lines.

### Grain size and weight are more heat-sensitive in *OsCG5* mutants

**Figure 5:**
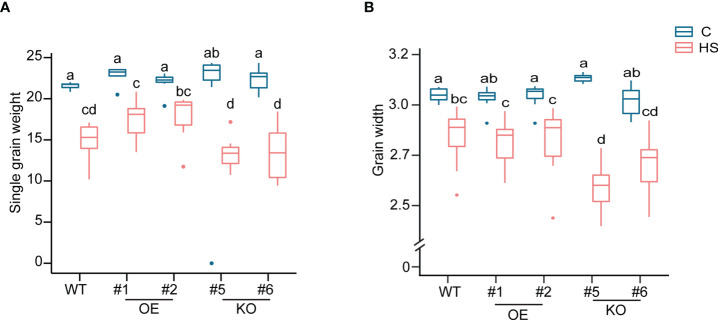
Morphometrics differences in single grain weight (SGW) and grain width from WT, KO and OE plants under control and heat stress. **(A)** SGW **(B)** Grain width. The significance level was estimated using two-way ANOVA. *N* = 6-7 plants. C and HS indicate control and heat stress, respectively.

A difference in the chalkiness among the grains from three genotypes prompted us to measure the grain size and weight from WT, OE and KO lines as higher grain chalkiness may lead to a reduction of grain size and weight due to the airspaces created among the abnormal starch granules. We found that HS caused a significant reduction in single grain weight (sgw), grain width, and grain area for all lines, but grain length showed minimum sensitivity to HS (**Figure 5**, **Figure S5**, **Table S6**). Notably, HS resulted in most reduction in grain size parameters for KO mutants. Grain length was not significantly impacted by HS for OE and WT. However, KO mutant grains also showed a reduction in grain length (17.39% and 10.9% for KO#5 and KO#6, respectively) compared to corresponding controls (**Table S6**). We also estimated the total yield for WT, OE and KO and found that HS caused a severe yield reduction in these genotypes. We did not observe a genotypic-specific significant difference in the total yield reduction (**Figure S5C**). However, KO#5 showed a maximum percentage reduction (50%) in total yield (**Table S6**). Collectively, these results suggest that developing grains deficient in *OsCG5* are more sensitive to HS imposed during early grain development regarding grain size and quality.

### Co-expression analysis links *OsCG5* function to endosperm transfer cell layer

We next sought to develop a probable regulatory pathway for *OsGC5* by performing an *in-silico* gene co-expression analysis at the early grain filling stage. We used a public transcriptome dataset for 2 and 3 DAF with three temperature treatments (28°C, 35°C and 39°C) to identify genes co-regulating with *OsCG5* (Chen et al., 2016). We found 1,653 positively and negatively (*PCC*>0.8 or *PCC*< -0.8 with *P*<0.05, respectively) co-regulating partners of *OsCG5* (**Table S7**). A subset of the positively co-regulated genes belongs to families such as maternally expressed genes (MEG), defensin, glycosyl hydrolase, invertase, lipid transfer protein (LTP), transferase, and Sugars Will Eventually be exported Transporters (SWEET), which have previously been shown to function in basal endosperm transfer layer (BETL) in maize (Lopato et al., 2014; Sosso et al., 2015; Salminen et al., 2016). For instance, *Maternally expressed gene* (MEG) *1* has specific expression in maize BETL cells (Gutiérrez-Marcos et al., 2004). Our analysis revealed that 7/11 rice MEG family genes (*MEGL3, MEGL5, MEGL8, MEGL11, MEGL12, MEGL13* and *MEGL19*) are positive co-regulated with *OsCG5*. Similarly, LTP family genes *OsPR602* and *OsPR9a* are highly expressed in endosperm transfer cells (ETC) during early grain filling stages in rice (Li et al., 2008). We found *OsPR602* to be co-regulated with *OsCG5* at the grain filling stage. These results suggest that *OsCG5* may interact with other genes expressed in ETC during the grain filling stage.

## Discussion

In this study, we show that rice grain RG values can be used as a quantitative estimator for chalkiness. Using RG values, we estimated the extent of natural variation in heat response of RDP1 accessions for grain chalkiness. Evaluation of grain chalkiness from RG values obtained from *SeedExtractor* is a low-cost, time-efficient, and non-destructive method that can be easily scaled to screen large germplasm. Our results show that grains with severe chalkiness tend to have higher RG values than translucent grains, suggesting a positive relationship between grain RG values and chalkiness levels (**Figure 1**). Using RG value as a proxy trait for grain chalkiness or quality, we performed GWA analysis and identified several novel loci associated with grain RG under control and HS with a probable role in grain chalkiness (**Figure 2**; **Table S5**). Grain chalkiness is not only triggered by high temperature but also determined by factors such as grain size and humidity level (Misra et al., 2021). For instance, a higher grain width with low or no amylose content leads to higher grain chalkiness. The RDP1 population used in the study consist of accessions with diverse grain size properties. As a result, we may see a difference in grain chalkiness of these accessions even under control at a minimal level. Therefore, significant SNPs detection under control and HS is expected depending on whether the natural variation contributes to the chalkiness in the respective environment. GWAS SNPs identified in this study co-localized with loci such as pentatricopeptide repeat domain, vacuolar ATP synthase subunit, and endoplasmic reticulum-Golgi intermediate compartment protein (*LOC_Os11g37330*, *LOC_Os05g40230*, and *LOC_Os05g41120*, respectively), which are known to regulate grain chalkiness. ER stress response is one of the early drivers of HS responses in grains. Mutants impaired in ER-pathway produce chalky/opaque grains in rice (Yasuda et al., 2009; Sandhu et al., 2021; Yang et al., 2022). We identified a prominent HS-specific peak on Chr 5 and narrowed down to a candidate (*OsCG5*) based on its tissue and temporal expression pattern. *OsCG5* transcripts are detected during the early grain development window that coincides with our HS treatment. Further, the transcript abundance of *OsCG5* in developing grains is sensitive to temperature increases. Along with HS response and grain-specific expression, our rationale for characterizing the *OsCG5* is also driven by the fact that expressed protein-coding genes are among the least explored class of genes in the rice genome due to the lack of information on the protein domains. Despite being given less attention, expressed proteins have been shown to have important roles during development. For instance, a class of expressed proteins such as microproteins have been shown to fine-tune an array of events, including shoot apical meristem maintenance and flowering time regulation (Bhati et al., 2020).

Based on allelic variation and transgenic studies, we present evidence that *OsCG5* regulates grain chalkiness under HS. The HS-treated grains from major allele accessions (Ma) for *OsCG5* SNP carrying ‘G’ allele had lower RG values and chalkiness than minor allelic accessions with ‘T’ allele (**Figure 3**). Allelic frequency indicates that the major allele for SNP-5.23896968 is predominant in RDP1. This distribution shows that the major allele possibly has undergone a positive selection during evolution, contributing to the grain quality. We show that allelic difference in grain RG could be a consequence of differential transcript abundance of *OsCG5* as Ma accessions generally had higher transcript abundance of *OsCG5* under control and HS compared to Mi accession (**Figures 3D** **, E**). It is also noteworthy that while the average transcript abundance of *OsCG5* was not significantly different under control and HS for Mi, we detected more than 3-fold induction of *OsCG5* transcripts in two major (Ma1 and Ma2) accessions under HS with a minimal increase in Ma3 (**Figure 3D**). Since, different rice accessions can have slightly varying grain developmental progression, it is possible that lower expression level on Ma3 could be due to such a difference. This is relevant for *OsCG5* as it is expressed for a short duration during early grain development and developmental progression during this stage is highly sensitive to HS. Overall, our analysis suggests that higher transcript abundance of *OsCG5* may contribute to lower chalkiness and hence lower HS sensitivity in the major allelic group (Ma).

This is supported by increased chalkiness of grains from KO lines under HS but not under control temperatures. This suggests that Kitaake *OsCG5* does not contribute to the chalkiness trait under normal temperatures. Overexpressing *OsCG5* in Kitaake did not decrease chalkiness under HS. This could be because the basal (control) level of *OsCG5* in the major alleles examined here (**Figure 3**) may be sufficient to limit chalkiness under HS. The observed marked increase under HS for lines Ma1 and Ma2 may be inconsequential with regards to chalkiness. These results corroborate the phenotypic difference found in allelic variants of *OsCG5* (**Figures 3**, **4**). We complemented the results obtained from transgenic studies using hyperspectral reflectance analysis of grains from different genetic backgrounds (**Figure 4E**). We found an increase in spectral reflectance of transgenic grains with increase in chalkiness. A clear separation of spectral lines identified for WT, KO, and OE grains at 1400-1650 nm is comparable to the various degrees of chalkiness observed in these genotypes. The comparison of RG values with hyperspectral scan of grains suggest that these two platforms are complementary and have different sensitivity in distinguishing the mutants from WT.

We evaluated our GWAS results by comparing them with novel QTLs or loci identified in previous association studies on grain chalkiness. Comparative analysis showed that three genes associated with significant SNPs identified in our study, RNA polymerase sigma factor, a hypothetical protein, and retrotransposon protein (*LOC_Os11g26160*, *LOC_Os05g37090*, and *LOC_Os05g37100*, respectively) have also been detected as candidate genes regulating chalkiness in the GWAS study of 583 accessions from *indica* and *japonica* panels and multi-parent advanced generation intercross populations (Misra et al., 2021). Similarly, detection of an expressed protein coding gene (*LOC_Os05g09200*) in the present study and a TGWAS study with a different population structure indicates the high probability of *LOC_Os05g09200* functioning as a regulator of chalkiness (Misra et al., 2019). We did not identify a significant overlap with other association studies on grain chalkiness, which may be due to the complexity in the genetic architecture of this polygenic trait. Since our HS treatments were imposed precisely for 5d during early grain development, which is normally not the set-up used by other studies especially in the field. Our experimental treatment choice may have increased the likelihood of identifying novel loci that have higher developmental specificity.

Our co-expression analysis indicates that *OsCG5* mediated grain chalkiness functions in the ETC layer or by interacting with other genes expressed in the ETC layer. We detected the promoter-GUS activity at the base of the developing endosperm, which is consistent with the expression of BETL genes in maize (**Figure 3B**). The rice ETC layer, the equivalent of maize BETL, channels nutrients from maternal tissues to developing endosperm and protects the grains from infection (Li et al., 2008). Having a role of ETC layer in pathogen defense, eight defensin family genes co-regulated with *OsCG5* may have a role in the biotic stress tolerance of developing grains. Two maize BETL genes (BETL-1 and BETL-3) show sequence homology with defensin-like proteins (Li et al., 2008). Most of the MEGs are exclusively expressed in the BETL region in maize (Xiong et al., 2014). Given the identification of 7 MEGs co-regulating with *OsCG5*, we speculate that some may share functional role with their maize orthologs.

## Summary

This study identifies the grain RG trait as a potential means to estimate HS induced grain chalkiness. Integrating RG values as a phenotypic trait in GWAS yielded a novel candidate *OsCG5*. Functional validation suggests that *OsCG5* may regulate natural variation for grain quality under HS. Transgenic studies further suggested that the transcript abundance of *OsCG5* positively regulates grain quality under HS. Given the grain quality reduction due to HS and frequent heat waves occurring more frequently, natural variants of *OsCG5* may serve as a potential genetic resource to mitigate the grain quality reduction in breeding programs. A similar functional characterization strategy might be required to reveal the role of other candidate genes identified in this study in regulating grain quality under HS.

## Data availability statement

The data presented in the study are included in the article/**Supplementary material**. Further inquiries can be directed to the corresponding author.

## Author contributions

HW and PP conceptualized the project. AKNC and JS lead the study. PP and BKD conducted the heat stress experiment on rice diversity panel accessions, and PP scanned the grains. JS and LI generated the mutants, overexpression, and promoter GUS lines. AKNC performed the heat stress experiment and scanning of transgenic grains. WH and GM conducted GWAS analysis. TG and HY conducted the hyperspectral image analysis. AKNC and JS wrote the manuscript. PS and HW critically evaluated and reviewed the work. All authors read and approved the manuscript.

## Funding

This work was supported by National Science Foundation under Grant No. 1736192 awarded to HW.

## Acknowledgments

We would like to thank Kyle Wallman and Yuvraj Chopra for their help in collecting and sorting marked grains. We would also like to thank Martha Rowe for critical evaluation of the manuscript.

## Conflict of interest

The authors declare that the research was conducted in the absence of any commercial or financial relationships that could be construed as a potential conflict of interest.

## Publisher’s note

All claims expressed in this article are solely those of the authors and do not necessarily represent those of their affiliated organizations, or those of the publisher, the editors and the reviewers. Any product that may be evaluated in this article, or claim that may be made by its manufacturer, is not guaranteed or endorsed by the publisher.

## Supplementary material

Supplementary Figure S1 Principal component analysis depicting population structure of rice diversity panel 1 accessions.

Supplementary Figure S2 Expression pattern of *chalky grain 5* in **(A)** various anatomical samples **(B)** developing grains (12 - 48 hours after fertilization) profiled from time-series RNASeq dataset (Sandhu et al., 2021). **C** and HS indicate control and heat stress, respectively.

Supplementary Figure S3 GUS-stained samples of **(A)** 1 week old seedling **(B)** germinating grain.

Supplementary Figure S4 RT-PCR assay showing the reduction in the expression of *chalky grain 5* in KO#5 and KO#6 transgenic lines compared to wild-type. Significance level for t-test, *, *P*<0.05; **, *P*<0.01.

Supplementary Figure S5 Morphometrics differences in grain area, grain length and total yield from WT KO and OE plants under control and heat stress. **(A)** Grain area **(B)** Grain length **(C)** Total yield. The significance level was estimated using two-way ANOVA. N = 6-7 plants. C and HS indicate control and heat stress, respectively.

Supplementary Table S1 Details of rice diversity panel 1 accessions used in the study.

Supplementary Table S2 List of all primers used in the study.

Supplementary Table S3 The number of grains used for each transgenic line for the phenotypic evaluation.

Supplementary Table S4 Distribution of the ratio of red to green pixels (RG) under control and heat stress (HS) along with their percentage change (HS/Control) among the selected accessions from rice diversity panel 1.

Supplementary Table S5 Details of significant SNPs and their associated locus (10kb upstream and downstream) under control and heat stress.

Supplementary Table S6 Percentage reduction (%) of various grain parameters in wild-type, knockout and overexpression lines under heat stress.

Supplementary Table S7 Genes positively and negatively co-regulated with OsCG5 identified from pairwise Pearson correlation analysis using the dataset from Chen et al., 2016.
