# Supplementary figures and images for: Rice Chalky Grain 5 regulates natural variation for grain quality under heat stress
Source: Front Plant Sci. 2022 Oct 11;13:1026472. doi: 10.3389/fpls.2022.1026472 (PMC9593041; doi:10.3389/fpls.2022.1026472)

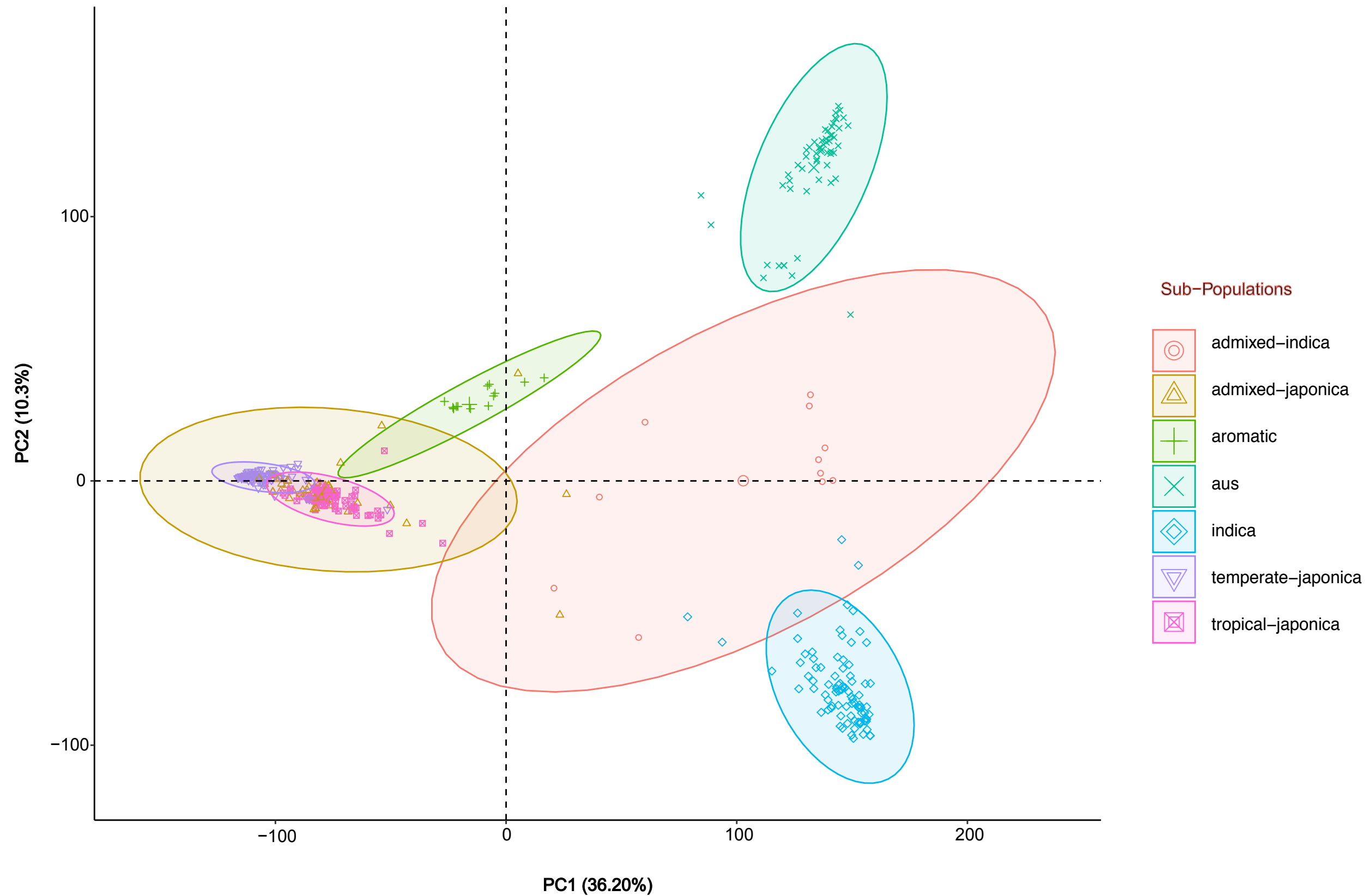

Supplement: Supplementary Figure S1 — Principal component analysis depicting population structure of rice diversity panel 1 accessions. [file DataSheet_1.pdf]

A)  
Expression atlas (GSE6893)

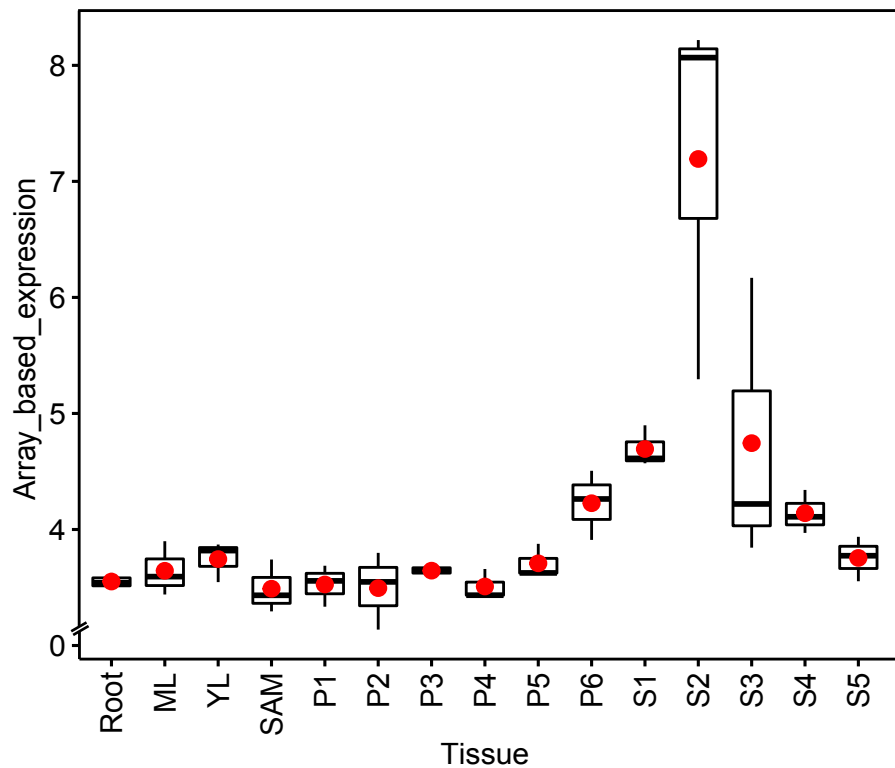

B)  
Time series grain developmental expression

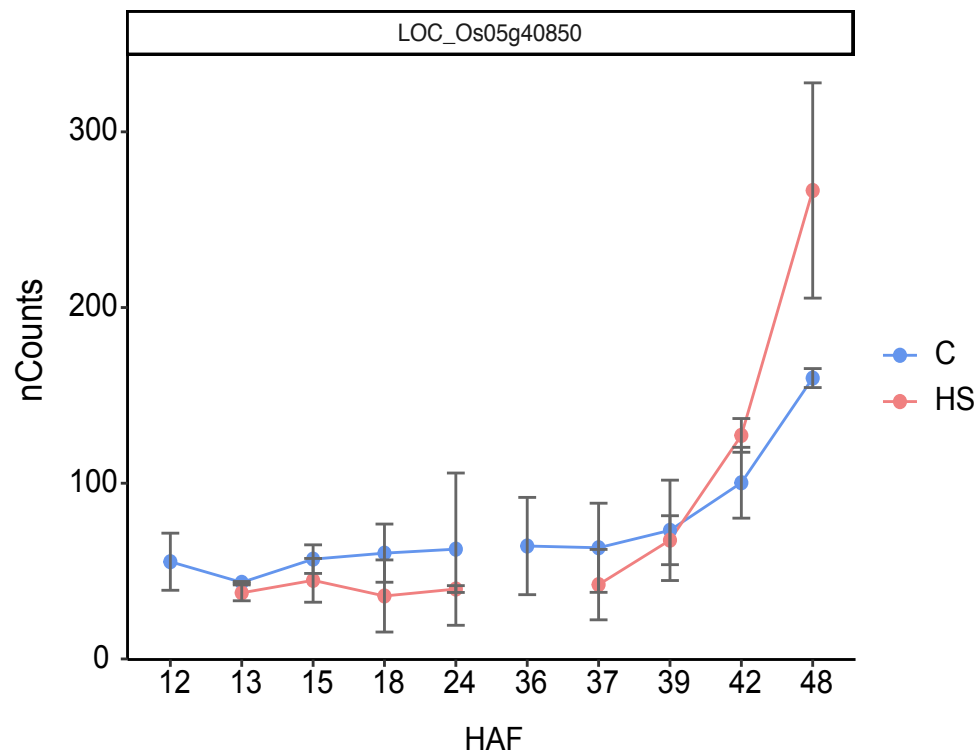

Supplement: Supplementary Figure S2 — Expression pattern of chalky grain 5 in (A) various anatomical samples (B) developing grains (12 - 48 hours after fertilization) profiled from time-series RNASeq dataset (Sandhu et al., 2021). C and HS indicate control and heat stress, respectively. [file DataSheet_2.pdf]

**A**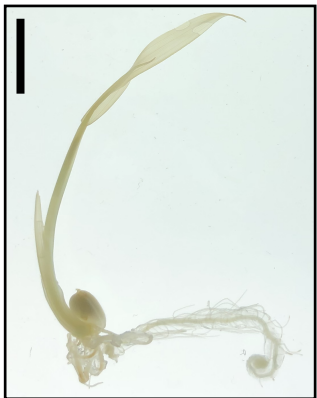**B**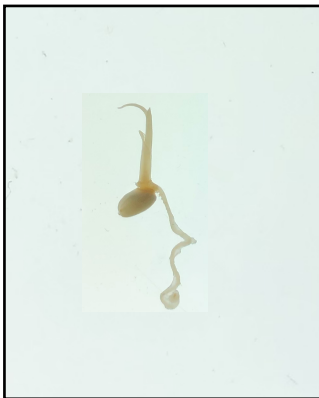

Supplement: Supplementary Figure S3 — GUS-stained samples of (A) 1 week old seedling (B) germinating grain. [file DataSheet_3.pdf]

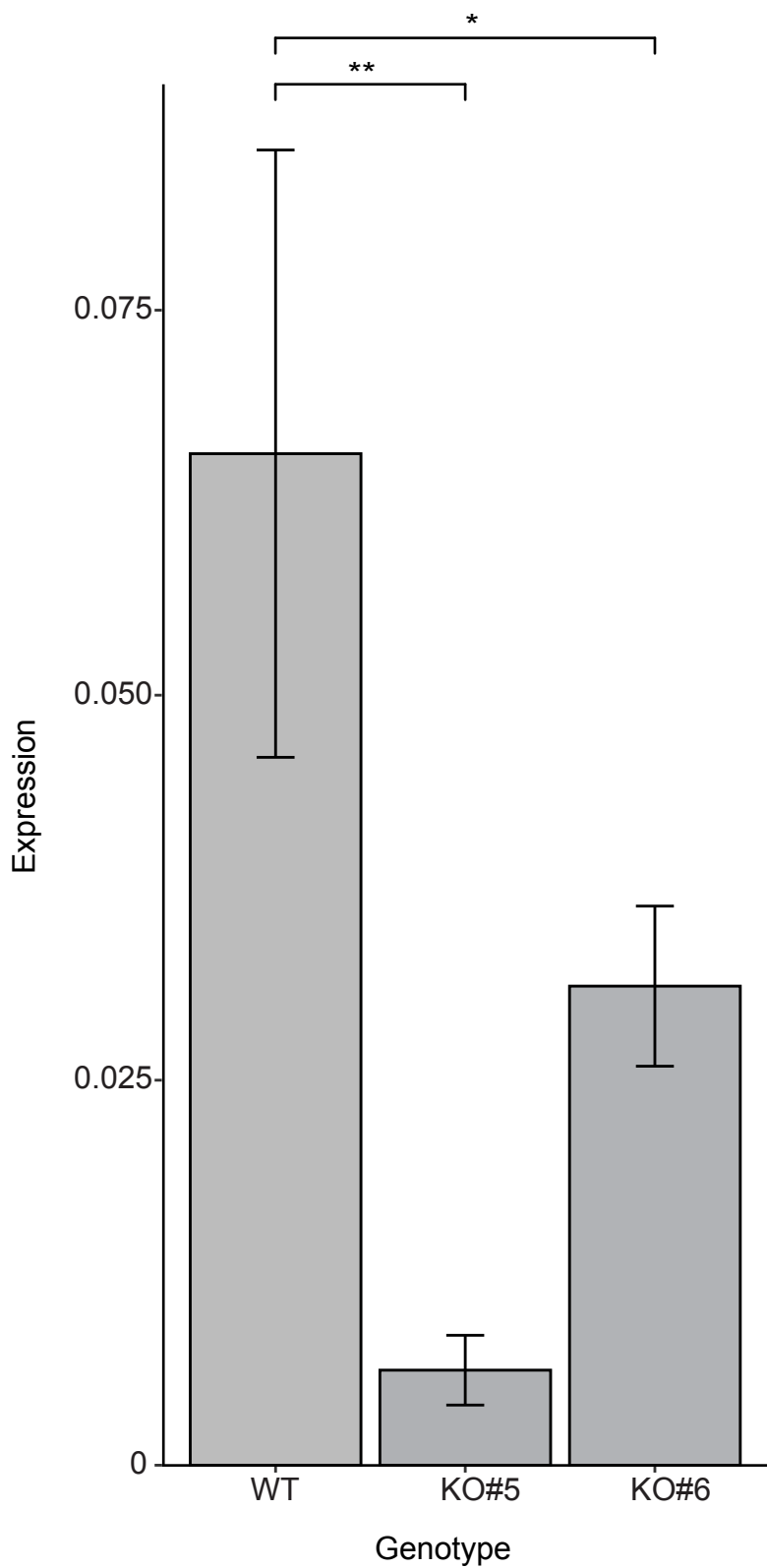

Supplement: Supplementary Figure S4 — RT-PCR assay showing the reduction in the expression of chalky grain 5 in KO#5 and KO#6 transgenic lines compared to wild-type. Significance level for t-test, *, P<0.05; **, P<0.01. [file DataSheet_4.pdf]

C  
HS

A

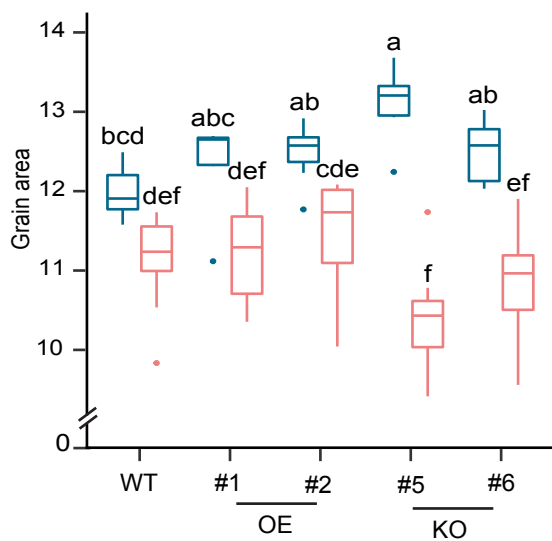

B

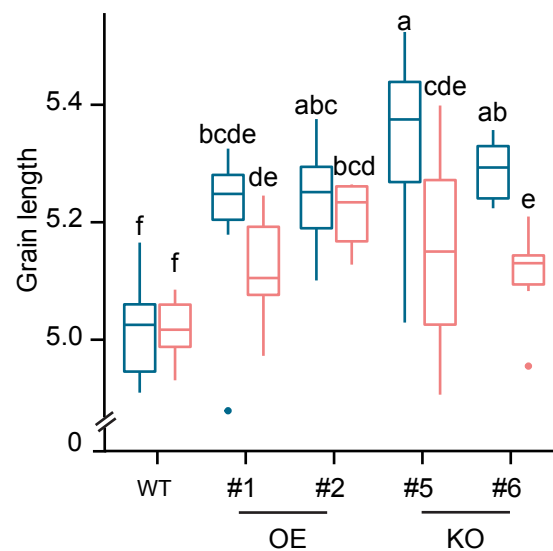

C

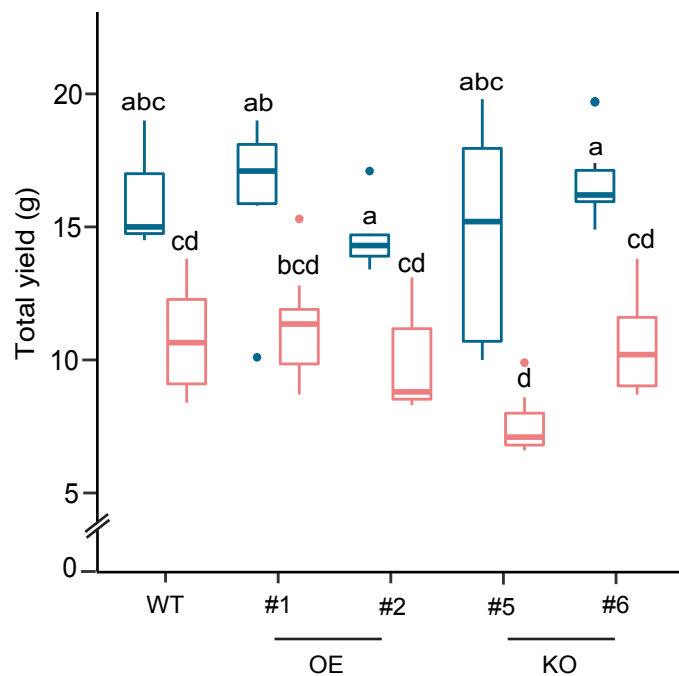

Supplement: Supplementary Figure S5 — Morphometrics differences in grain area, grain length and total yield from WT KO and OE plants under control and heat stress. (A) Grain area (B) Grain length (C) Total yield. The significance level was estimated using two-way ANOVA. N = 6-7 plants. C and HS indicate control and heat stress, respectively. [file DataSheet_5.pdf]
